# Comparing the importance of quality measurement themes in juvenile idiopathic inflammatory myositis between patients and families and healthcare professionals

**DOI:** 10.1186/s12969-018-0248-7

**Published:** 2018-04-19

**Authors:** Heather O. Tory, Ruy Carrasco, Thomas Griffin, Adam M. Huber, Philip Kahn, Angela Byun Robinson, David Zurakowski, Susan Kim, the CARRA Juvenile Dermatomyositis Quality Measures Workgroup

**Affiliations:** 1 Pediatric Rheumatology, Connecticut Children’s Medical Center, 282 Washington Street, Hartford, CT 06106 USA; 2 Department of Pediatrics, University of Connecticut School of Medicine, 263 Farmington Avenue, Farmington, 06032 CT USA; 3 Pediatric Rheumatology, Dell Children’s Medical Center of Central Texas, 4900 Mueller Boulevard, Austin, TX 78723 USA; 4 Pediatric Rheumatology, Carolinas HealthCare System, Levine Children’s Hospital Specialty Center, 1781 Tate Boulevard, SE, Suite 206, Hickory, NC 28054 USA; 5 Pediatric Rheumatology, IWK Health Centre and Dalhousie University, 5980 University Avenue, Halifax, NS B3K 6R8 Canada; 6 Pediatric Rheumatology, New York University Medical Center, 160 E 32nd Street, New York, NY 10016 USA; 7 Pediatric Rheumatology, Rainbow Babies & Children’s Hospital, 11100 Euclid Avenue, Cleveland, OH 44106 USA; 8 Departments of Anesthesia and Surgery, Boston Children’s Hospital and Harvard Medical School, 300 Longwood Avenue, Boston, MA 02115 USA; 9 Pediatric Rheumatology, Benioff Children’s Hospital and University of San Francisco Medical Center, 1975 4th Street, San Francisco, CA 94158 USA

**Keywords:** Juvenile dermatomyositis, Quality measures, Physician perspective, Patient perspective, Patient reported outcomes

## Abstract

**Background:**

A standardized set of quality measures for juvenile idiopathic inflammatory myopathies (JIIM) is not in use. Discordance has been shown between the importance ascribed to quality measures between patients and families and physicians. The objective of this study was to assess and compare the importance of various aspects of high quality care to patients with JIIM and their families with healthcare providers, to aid in future development of comprehensive quality measures.

**Methods:**

Surveys were developed by members of the Childhood Arthritis and Rheumatology Research Alliance (CARRA) Juvenile Dermatomyositis Workgroup through a consensus process and administered to patients and families through the CureJM Foundation and to healthcare professionals through CARRA. The survey asked respondents to rate the importance of 19 items related to aspects of high quality care, using a Likert scale.

**Results:**

Patients and families gave generally higher scores for importance to most of the quality measurement themes compared with healthcare professionals, with ratings of 13 of the 19 measures reaching statistical significance (*p* < 0.05). Of particular importance, however, was consensus between the groups on the top five most important items: quality of life, timely diagnosis, access to rheumatology, normalization of functioning/strength, and ability for self care.

**Conclusions:**

Despite overall differences in the rating of importance of quality indicators between patients and families and healthcare professionals, the groups agreed on the most important aspects of care. Recognizing areas of particular importance to patients and families, and overlapping in importance with providers, will promote the development of standardized quality measures with the greatest potential for improving care and outcomes for children with JIIM.

## Background

The juvenile idiopathic inflammatory myopathies (JIIM) represent a group of rare conditions with the common feature of muscle weakness, the most frequent of which is juvenile dermatomyositis (JDM). These are chronic conditions, often associated with long-term morbidity due to the disease itself and medication toxicities, which can lead to significant functional impairment. While there are well documented clinical and laboratory criteria for assessment and ongoing monitoring of disease activity in patients with JIIM, and published core sets of outcome measures including disease activity and damage assessment [1, 2], there is currently no standardized, comprehensive set of quality measures for monitoring disease activity, disease chronicity, response to therapy and functional impact in patients with JIIM in clinical practice.

Quality measures have been developed and adopted largely to enable the evaluation of the quality and performance of healthcare delivery by a healthcare provider or entity. They facilitate standardized comparisons of clinical care across care providers and are becoming increasingly important in assessing healthcare delivery processes and outcomes [3]. At the level of the individual clinician, they should serve as a roadmap to delivering the highest quality care for patients and represent the standard to achieve for every patient.

The Patient-Reported Outcomes Measurement Information System (PROMIS) measures are quickly becoming standards in evaluation of clinical practice monitoring for a multitude of disease processes, including the rheumatic diseases [4]. Involving patients in their own care significantly improves healthcare outcomes, healthcare utilization and patient satisfaction [5]; however, most patient reported outcomes (PROs) have been developed without significant patient input. Standard sets of quality measures to guide clinical care have been established for some pediatric rheumatic diseases, but have not been widely used or well defined for JIIM [6, 7].

The development of quality measures in JIIM, with a focus on PROs, is critical for giving medical providers the tools to monitor and treat patients with the disease in a manner that reflects the desired health outcomes of patients. This is also important for providing patients, parents, and caregivers the tools and information they need to make informed decisions about their healthcare. At the same time, the correlation between patient-reported and physician-reported outcome measures is not clearly established, with some studies suggesting significant discordance [8–10].

In this study, we sought to assess the importance of various aspects of high quality care to patients with JIIM and their families and assess the concordance with the importance ascribed by physicians and other clinical care providers. The ultimate goal of this work is to develop a standardized set of quality measures for use by pediatric rheumatologists and health professionals to aid in the assessment and long-term monitoring of patients with this disease.

## Methods

### Patient and family survey

A survey was developed by members of the Childhood Arthritis and Rheumatology Research Alliance (CARRA) JDM Quality Measures Workgroup through a consensus process. First, a list of candidate themes important in the clinical care of patients with JIIM was generated in round robin fashion, based on discussion among the ten workgroup members (including pediatric rheumatology attending physicians and trainees as well as allied health professionals) at an annual CARRA meeting. This was followed by selection of a draft list to include on the survey, using nominal group technique. Following the workgroup session, the survey underwent review and editing for additional input, suggestions regarding missing items, and evaluation of comprehensibility/readability by three parent members of the CureJM Foundation, a nonprofit organization dedicated to enhancing awareness and raising research funds for juvenile myositis. The final included list of variables then underwent an additional review online by the workgroup members for final approval.

The survey questions gathered information on patient demographics, myositis characteristics, functional disability, and asked respondents to rate the importance of 19 aspects of high quality care: timely diagnosis, access to rheumatology (ease of getting an appointment), access to dermatology, access to physical therapy, medication counseling, monitoring of medication toxicity, medication tolerance, discontinuation of steroids/prednisone, discontinuation of medications, normalization of labs, overall quality of life, ability for self-care/activities of daily living, resolution of pain, resolution of fatigue, resolution of rash, normalization of functioning/strength, school attendance, work attendance, and minimizing hospital/clinic visits. For these questions, respondents were asked to rank each measure independently on a 5-point scale, with “1” indicating very low importance and “5” indicating the greatest importance.

In June 2014, the survey was distributed electronically via e-mail to patients and family members of patients with JIIM through the CureJM Foundation patient and family database (https://www.surveymonkey.com/r/JM_QI). This database included contact information for approximately 1500 families of patients with JIIM. Responses were collated and data were abstracted in a standardized database for analysis. IRB approval was obtained at one institution and participants were informed that consent to participate was implied by completion of the questionnaire.

### Healthcare professional survey

The survey sent to patients and families of patients with JIIM was adapted for use with healthcare professionals. The survey questions assessed characteristics of the responding healthcare professionals and asked respondents to rate the importance of the same 19 quality of care themes. The survey was electronically distributed via e-mail to all registered CARRA healthcare professionals in the CARRA database (approximate *n* = 400, including physicians, nurses and other allied health professionals) in June 2015, with an additional reminder in July 2015 (https://www.surveymonkey.com/r/9WG3WWB). These responses were collated and data were abstracted in the same standardized database for analysis. IRB approval was obtained at one institution and participants were informed that consent to participate was implied by completion of the questionnaire.

### Statistical analysis

Student t-test comparisons were used to assess difference in the ranking of quality themes between parents and families and healthcare professionals. We used 2-tailed Student t-tests with *p*-values based on equal or unequal variances, as assessed by Levine’s test. Two-tailed values of *p* < 0.05 were considered statistically significant. Analysis was performed using IBM SPSS Statistics version 23.0 (IBM Corporation, Armonk, NY).

## Results

### Patient and family survey

Overall, there were 194 respondents (approximate response rate of 13%) to the survey, the majority of whom were parents of children with JIIM and the remainder of whom were mainly other relatives acting as caregivers. Most respondents described significant functional impact when the disease was at its worst, but currently had well-controlled disease, with minimal or no impact within the preceding two weeks (Table 1).

**Table 1:** Demographic information of patients represented by respondents to the patient and family survey

Characteristic	All patients (*N* = 194)
Diagnosis	
Juvenile dermatomyositis, N (%)	189 (97)
Juvenile polymyositis, N (%)	5 (3)
Respondent^a^	
Parent, N (%)	168 (87)
Grandparent, N (%)	10 (5)
Aunt/Uncle, N (%)	2 (1)
Female, N (%)	140 (72)
Ethnicity, N (%)	
Caucasian/White	150 (77)
Hispanic/Latino	10 (5)
African American/Black	11 (6)
Asian/Pacific Islander	2 (1)
American Indian/Alaskan Native	1 (0.5)
Multiple Ethnicity	18 (9)
Prefer not to answer	1 (1)
Time to diagnosis (months): mean (range)	6.7 (1–40)
Functional disability when disease at worst	
Significant impact, N (%)	145 (75%)
Intermediate impact, N (%)	24 (12%)
Functional disability in prior two weeks	
Minimal or no impact, N (%)	131 (69%)
Significant impact, N (%)	19 (10%)

^a^Total less than 100%: 14 (7%) of respondents skipped question

**Fig. 1:**
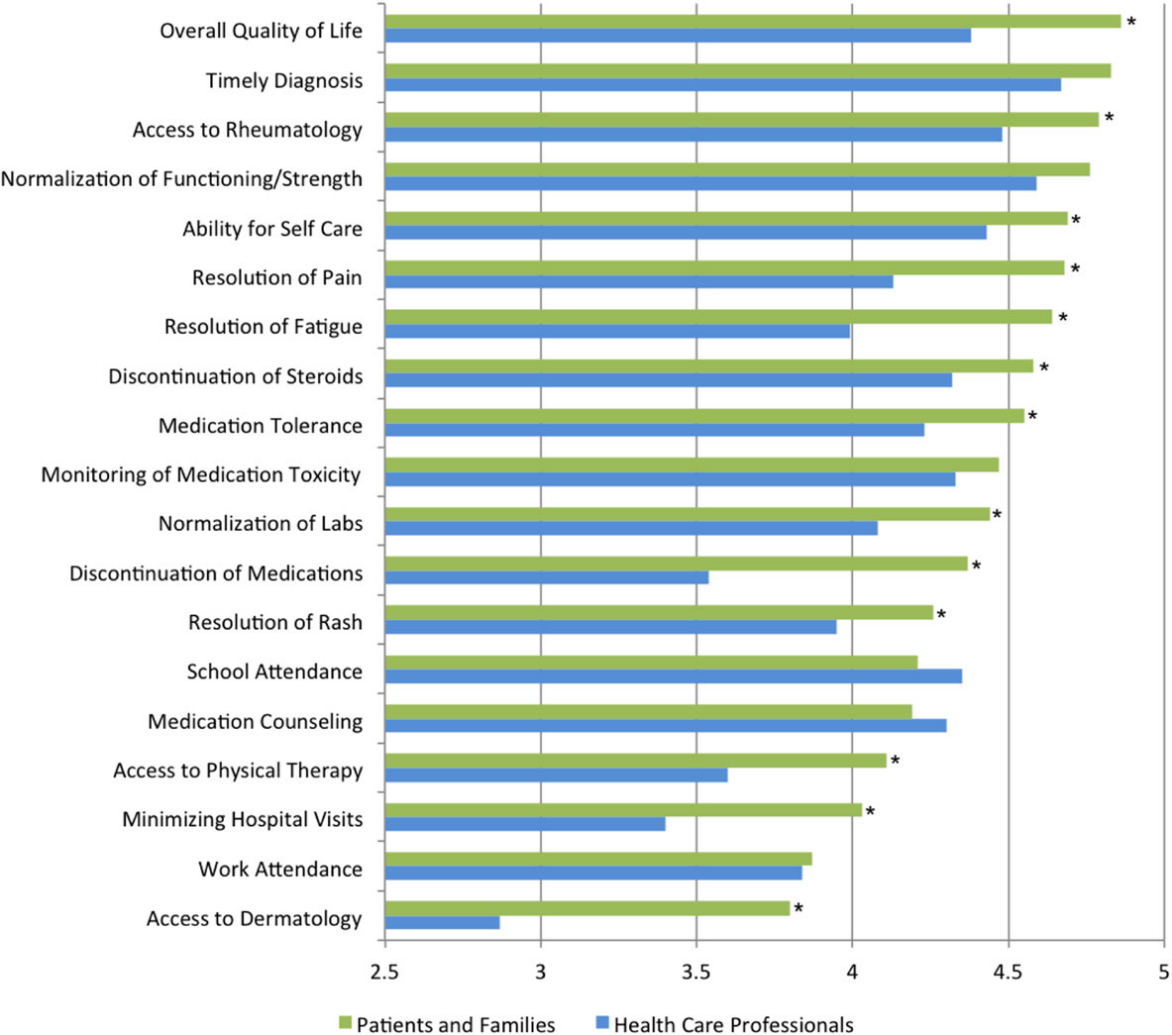
Relative rating of quality measurement themes to patients and families of patients with JIIM and healthcare professionals caring for patients with JIIM. *Denotes statistical significance of *p* < 0.05 using Student t-test

Families of patients with JIIM rated overall quality of life as the variable with the highest average importance. Timely diagnosis and access to rheumatology were the next most important, followed by patient reported outcomes of normalization of functioning and strength, resolution of pain, and resolution of fatigue. Conversely, access to a dermatologist and concerns related to work attendance and number of hospital and clinic visits, were rated as least important (Fig. 1); however, all of the items were highly rated, with the lowest ranked (access to dermatology) receiving an average score of 3.8 out of five.

In subgroup analysis, there was no significant difference in rating of quality themes when comparing patients diagnosed in the past two years, compared to patients diagnosed more than 2 years ago, except for normalization of labs (mean rating 4.6 +/− 0.755 vs. 4.3 +/− 1.136, respectively, *p* = 0.01). There were also no significant differences found in the ratings between patients reporting high and low levels of current functional limitations.

### Healthcare professional survey

There were 86 responses (approximate response rate of 22%) to the healthcare professional survey: 82 (95%) pediatric rheumatologists, 2 (2.5%) pediatric nurse practitioners, and 2 (2.5%) adult rheumatologists, all practicing in the United States (89.5%) and Canada (10.5%). Respondents were evenly divided among clinicians with 1–5 years of experience (25%), 6–10 years (22.6%), 11–20 years (23.8%) and more than 20 years (28.6%). Most respondents reported following less than 15 patients (80.7%), with only seven respondents (8.4%) reporting following more than 30 patients with JIIM.

Healthcare professionals rated timely diagnosis as the most important aspect of high quality care, followed by normalization of functioning/strength and access to rheumatology (Fig. 1).

### Patient and family and healthcare professional survey comparison

While there were differences observed in the relative ratings of the importance of various quality indicators between patients and families and healthcare professionals (Fig. 1), the groups agreed on the top five most important themes (Table 2). Overall, patients and families gave higher rankings than healthcare professionals to all of the variables except school attendance and medication counseling, although this difference was not statistically significant. There were 13 quality themes that parents rated as statistically significantly more important than healthcare providers. These areas were overall quality of life, access to a rheumatologist, ability for self care, resolution of pain and fatigue, discontinuation of steroids, medication tolerance, normalization of labs, discontinuation of medications, resolution of rash, access to physical therapy, minimization of hospital visits, and access to dermatology (all *p* < 0.05) (Fig. 1).

**Table 2:** Top five quality measurement themes rated as being most important by both patients and families with JIIM and healthcare professionals

Patients and Families	Healthcare Professionals	Top Five Quality Measurement Themes
1	5	Overall Quality of Life
2	1	Timely Diagnosis
3	3	Access to Rheumatology
4	2	Normalization of Functioning/Strength
5	4	Ability for Self Care

## Discussion

Healthcare providers and systems are increasingly focused on patient centered care, with the ultimate goal of improving care in a manner most relevant to patients. This study was performed to identify and compare the importance of various aspects of high quality care to patients and families with JIIM and their healthcare providers, to help guide the future development of a standardized set of clearly defined quality measures that are relevant to both clinical care providers and patients and their families. This is an area of great need in JIIM, as standardized quality measures are important for improving the assessment and long-term monitoring of patients with chronic diseases [11]. While collecting an exhaustive list of measures would be comprehensive, it is impractical. With limitations in economic resources and time, identifying areas for development of quality measures with overlapping importance to patients and healthcare providers will highlight the best areas for initial focus.

Previous work completed by the Outcome Measures in Rheumatology (OMERACT) myositis working group, and others, has also shown the importance of patient input in the development of PROs in myositis [12]. Using qualitative studies with focused interview and cognitive debriefing methodologies to inform their work, this group found that currently used PROs do not measure the outcomes that are most important to patients. Similarly, our work also revealed differences between patient and provider perspectives in the ratings of importance of different variables, such as minimizing hospital visits and access to other health care professionals. Importantly, however, our study found that both groups mutually agreed upon the top five aspects of high quality care. This suggests that creating a standardized set of quality measures with relevance and meaning to patients and families and healthcare professionals is feasible and appropriate. These items are important to pursue in further quality measurement development, and may be the cornerstone in defining a standardized set of quality measures in JIIM.

There are limitations to consider in our work. The patient and family and healthcare professional surveys did not undergo formal reliability or validity testing, as they were exploratory in nature. The healthcare provider survey was sent to the entire CARRA membership, with a response rate of approximately 22%. While this rate can be considered low, it is similar to the response rates in other recent CARRA surveys [13] and is comparable to currently reported online survey response rates [14]. This rate is lower than that reported in some earlier surveys of CARRA work [15, 16]. The current survey was sent to the entire CARRA membership, not a specific membership subset as with some of these prior surveys (such as only to the JDM disease subcommittee), so it may be that clinicians with more exposure and expertise in JIIM were more likely to respond. With the limited response rate, it is possible that these findings are not representative of all CARRA members; however, there was broad representation of healthcare professionals, with a similar proportion of responses across all years of experience.

The patient and family survey was sent to members of the CureJM Foundation, which may represent a specific demographic population rather than the entire population of patients and families with JIIM. For example, the majority of patients appeared to be in remission, with relatively low disease impact at the time of the survey. The survey was also administered solely in English, which likely excluded a subset of patients. Furthermore, the majority of respondents were parents, not patients, which may alter the perspective and responses as well, although this is common in pediatrics. This was likely due to greater parent membership in the CureJM Foundation, compared with patients. Finally, the quality measurement themes that were presented for ranking of importance were selected and incorporated into the survey by healthcare providers and three parent reviewers, but there may be other measures that are important to patients and families that they were not given the opportunity to rank. This would benefit from additional input from a broader patient and family audience through focus groups and other methods of communication.

In general, the overall rankings of importance of all the variables were scored higher by patients and families compared to healthcare providers. Although this could be an accurate measurement, it may reflect the differences in perspective when comparing patients’ and providers’ experiences and goals. It is also likely that healthcare professionals are more accustomed to scales/rankings, leading to a larger range of rankings when compared to patients and families. This may have been minimized if the ratings were measured in an ordinal scale, which should be applied to future work. In addition, these differences may be related to the finding that patients and families are more likely to use temporal comparisons (considering these measures based on their personal condition), while clinicians appear to use social comparisons (considering measures based on the full range of disease in the population) [17].

We had hypothesized that there might be differences in the ranking of importance of all of the themes based on duration of disease or current level of functional impairment from disease among patients and families, but this was not shown in statistical analysis. This may have been due to the relatively low percentage of patients with any current functional impact, as nearly 70% of respondents reported minimal or no current functional impact from JIIM at the time of their response to the survey. Access to dermatology was rated lowest by patients and families compared to other items and may have been influenced by the fact that the survey was administered by a group of rheumatologists through CARRA.

Healthcare professionals must be thoughtful in aligning standardized quality measures with those that are considered important by patients and their families, to ensure that the patient voice and perspective is being incorporated into decision making for disease assessment, monitoring and treatment. Attention to quality measures that are important to patients and their families will also promote partnership in care and alignment of goals between patients and care providers which may, in turn, help to improve adherence to therapy, satisfaction, and outcomes. Understanding and appreciating differences in the perceptions of relative importance of the aspects of quality care between these groups should be considered in future plans to design and implement a standardized set of quality measures for this disease, with particular attention paid to those areas that showed overlapping highest importance (quality of life, timely diagnosis, access to rheumatology, normalization of functioning/strength, and ability for self care). With limited resources, the integration of measures that are considered important by both patients and families and clinicians may allow for future pragmatic collection of high-yield outcome measures, and maximize the opportunities to measure and improve outcomes for children with JIIM. To move this work forward and reach that goal, the themes discussed here will need to be explored and transformed into clearly developed quality measures with robust measurement specifications. This will require discussion of the measurement domains and methods, as well as the tools and methodology that are required to assess the variables. Some of the quality of care themes explored here will more readily translate into specific quality measures for use by individual pediatric rheumatologists. For example, quality of life can be assessed with the use of validated tools [18], while timely diagnosis is influenced by a variety of factors other than the care of the treating pediatric rheumatologist, as many delays in care are experienced prior to referral to rheumatology. This will need to be taken into account when designing specific, measurable parameters for quality measurement.

## Conclusions

The results of this study support prior work showing differences in the relative importance that patients and families ascribed to various quality measures, compared with healthcare providers. This highlights the importance of obtaining input and feedback from patients and families when designing and implementing quality measures for use in monitoring disease activity, progress and response to treatment. This study found that the top five themes for quality care ranked by patients and families and healthcare professionals were overlapping, suggesting that these domains should be given special attention and incorporated into future quality measure development for JIIM.

## Abbreviations

CARRA: Childhood Arthritis and Rheumatology Research Alliance
JDM: Juvenile Dermatomyositis
JIIM: Juvenile idiopathic inflammatory myopathies
OMERACT: Outcome Measures in Rheumatology
PROMIS: Patient-Reported Outcomes Measurement Information System
